# Socioeconomic inequalities in risk factors for non communicable diseases in low-income and middle-income countries: results from the World Health Survey

**DOI:** 10.1186/1471-2458-12-912

**Published:** 2012-10-28

**Authors:** Ahmad Reza Hosseinpoor, Nicole Bergen, Anton Kunst, Sam Harper, Regina Guthold, Dag Rekve, Edouard Tursan d'Espaignet, Nirmala Naidoo, Somnath Chatterji

**Affiliations:** 1 Department of Health Statistics and Information Systems, World Health Organization, 20, Avenue Appia, Geneva, CH-1211, Switzerland; 2 Department of Public Health, AMC, University of Amsterdam, Amsterdam, Netherlands; 3 Department of Epidemiology, Biostatistics & Occupational Health, McGill University, Montreal, Canada; 4 Department of Chronic Diseases and Health Promotion, World Health Organization, Geneva, Switzerland; 5 Department of Mental Health and Substance Abuse, World Health Organization, Geneva, Switzerland; 6 Tobacco Free Initiative, World Health Organization, Geneva, Switzerland

**Keywords:** Developing countries, Socioeconomic factors, Chronic disease, Risk factors

## Abstract

**Background:**

Monitoring inequalities in non communicable disease risk factor prevalence can help to inform and target effective interventions. The prevalence of current daily smoking, low fruit and vegetable consumption, physical inactivity, and heavy episodic alcohol drinking were quantified and compared across wealth and education levels in low- and middle-income country groups.

**Methods:**

This study included self-reported data from 232,056 adult participants in 48 countries, derived from the 2002–2004 World Health Survey. Data were stratified by sex and low- or middle-income country status. The main outcome measurements were risk factor prevalence rates reported by wealth quintile and five levels of educational attainment. Socioeconomic inequalities were measured using the slope index of inequality, reflecting differences in prevalence rates, and the relative index of inequality, reflecting the prevalence ratio between the two extremes of wealth or education accounting for the entire distribution. Data were adjusted for confounding factors: sex, age, marital status, area of residence, and country of residence.

**Results:**

Smoking and low fruit and vegetable consumption were significantly higher among lower socioeconomic groups. The highest wealth-related absolute inequality was seen in smoking among men of low- income country group (slope index of inequality 23.0 percentage points; 95% confidence interval 19.6, 26.4). The slope index of inequality for low fruit and vegetable consumption across the entire distribution of education was around 8 percentage points in both sexes and both country income groups. Physical inactivity was less prevalent in populations of low socioeconomic status, especially in low-income countries (relative index of inequality: (men) 0.46, 95% confidence interval 0.33, 0.64; (women) 0.52, 95% confidence interval 0.42, 0.65). Mixed patterns were found for heavy drinking.

**Conclusions:**

Disaggregated analysis of the prevalence of non-communicable disease risk factors demonstrated different patterns and varying degrees of socioeconomic inequalities across low- and middle-income settings. Interventions should aim to reach and achieve sustained benefits for high-risk populations.

## Background

The burden of non communicable diseases (NCDs) and associated risk factors is evident worldwide. The negative effects of globalization, rapid urbanization, sedentary lifestyles, and poor dietary habits, together with trends of population aging, constitute considerable challenges for governments and public health stakeholders [1]. This is especially true in low- and middle-income countries (LMICs) and among those living in poverty [1-3]. NCDs are increasingly affecting developing countries at a faster rate than in developed nations, [3] with about 80% of NCD-related deaths now occurring in LMICs [1,4].

Health-damaging behaviors such as smoking, heavy alcohol drinking, inadequate fruit and vegetable consumption, and physical inactivity are leading risk factors that contribute to multiple health conditions and diseases [1,5,6]. Although many lifestyle factors are considered to be modifiable, all individuals, across all strata of society, should have sufficient and equal access and support to make healthy lifestyle choices [5]. The World Health Organization (WHO) *Global status report on non communicable diseases 2010*[1] ranked the monitoring and surveillance of risk factors as a top priority to tackle growing NCD epidemics in low-resource settings. Increasingly, public health is turning to social determinants of health to explain health outcome inequalities [7-9].

Rates of health risk factors show patterns of considerable variability within developing countries, and may be unequally experienced by socioeconomic groups [10]. Populations that are socioeconomically disadvantaged in terms of education or income tend to fare worse with regards to NCD risk factor prevalence, [1] though important exceptions exist [11,12]. Some epidemiological evidence suggests earlier adoption of health risk behaviors by advantaged socioeconomic groups has been followed by increased prevalence among disadvantaged socioeconomic groups [13,14]. For example, Marins et al. [15] demonstrated a strong, inverse association of education level with an index of cardiovascular risk factors in Brazil.

Cross-national comparisons of health risk factors are useful in helping to uncover factors that work at national levels, and to characterize patterns of NCD risk factor distribution. Successful targeted NCD prevention efforts rely on the early detection of at-risk individuals [16]. National prevalence rates of NCD risk factors have recently been documented around the world (including LMICs), drawing from multiple data sources [17]. However, comparable international data about socioeconomic inequalities in health risk factors are scarce especially in LMICs.

## Methods

### Study population and ethics statement

Data for 50 LMICs were obtained from World Health Survey (WHS), carried out in 2002–2004. Conducted by the WHO, the WHS is a source of internationally-comparable population health information [18]. Analyses included 232,056 adult participants from the 48 countries (21 low-income countries (LICs) and 27 middle-income countries (MICs)) that had available data about NCD risk factors, and relevant socioeconomic and demographic variables. Guatemala did not have data on survey sampling weight, and Turkey had insufficient data to create the household wealth index, one of the principal variables of the study. Additional file 1 displays study sample size, by country and sex.

Household and individual questionnaires gathered data about household wealth, socio-demographics and health-related risk factors from respondents aged 18 years or higher. For physical inactivity, the survey instrument (International Physical Activity Questionnaire) was tested for validity for adults between ages 18 and 69, and thus analysis for this risk factor was limited to this age bracket [19]. The samples were nationally representative except in China, Comoros, Congo, Côte d'Ivoire, India, and the Russian Federation, where the WHS was carried out in geographically limited regions. The response rates were reported in two steps: household level and individual level. Response rates at the household level were over 70% in all countries except Congo (63.6%), Swaziland (53.8%) and Czech Republic (23.9%). Individual level response rates were above 82% [20]. Additional file 2 shows non-response rates for each risk factor, by country income group and sex.

Data were divided into four pooled datasets according to sex and LIC or MIC status. Countries were classified according to 2003 World Bank development categories, consistent with the timing of the majority of the WHS surveys [21]. Datasets that reported no heavy episodic alcohol drinkers were excluded from the analysis for that risk factor: male data from Mauritania, and female data from Bosnia-Herzegovina, Comoros, Mauritania and Pakistan. For both sexes, data about low fruit and vegetable diet were not available for Mexico, and physical inactivity data were not available for Latvia and Morocco.

Informed consent was obtained in all surveys. A standard consent form approved by the ethics review committee was read to the respondent in the respondent’s language. If the respondent was literate and gave consent to participate, the form was provided to the respondent to read and sign, and was countersigned by the interviewer. If the respondent was illiterate and gave consent to participate, the interviewer confirmed this consent by signing that the respondent had been read the form, understood the study, and agreed to participate. This procedure was approved by the institutional review boards in each study country. The full list of local review boards from each study country is available in Additional file 3.

### Variables

Four NCD risk factors were considered: current daily smoking, low fruit and vegetable consumption, physical inactivity, and heavy episodic alcohol drinking. *Current daily smoking* was the current daily use of any tobacco product including cigarettes, cigars, pipes, and local tobacco products [22]. *Low fruit and vegetable consumption* referred to an intake of fewer than five total servings of fruits and/or vegetables (about 400 grams) per day, [23] the general recommendation by the WHO panel on diet, nutrition and chronic disease prevention [24,25]. *Physical inactivity* was classified as the failure to meet WHO recommendations on physical activity for health, which are defined as engaging in at least 150 minutes of moderate-intensity activity per week or 75 minutes of vigorous-intensity activity per week, or an equivalent combination achieving a minimum of 600 MET-minutes per week (one MET-minute is equivalent to [4.184 kJ] ∙ kg ^-1^∙ h ^-1^) through any combination of walking and moderate- or vigorous-intensity activities [26]. *Heavy episodic alcohol drinking* was the consumption of five or more (in men) or four or more (in women) standard alcoholic drinks on at least one day during the preceding week [27].

Socioeconomic status was derived from individual household wealth status and highest-attained level of education. To measure household wealth, a dichotomous hierarchical ordered probit model was used to develop an index of the long-running economic status of households based on owning selected assets and/or using certain services [28-30]. The index was divided into five quintiles within each country, with quintile one representing the poorest wealth quintile and quintile five, the richest. Education was ranked according to five categories: no formal schooling, less than primary school, primary school completed, secondary/high school completed, and college completed or above.

Confounders included sex, age (expressed categorically as 18–29, 30–39, 40–49, 50–59, 60–69, or 70 or more years), marital status (married/cohabiting, divorced/separated/widowed, or never married), area of residence (rural or urban), and country of residence.

### Statistical analysis

Both age-standardized [31] and crude prevalence rates for each risk factor were calculated for men and women in LIC and MIC groups as overall prevalence, and according to wealth quintile and education level. Socioeconomic inequality in each risk factor prevalence was measured using the slope index of inequality (SII) and the relative index of inequality (RII), measures that account for population distribution across wealth quintiles or education levels [32]. A Poisson regression model with a robust variance was used to generate prevalence rate difference and prevalence rate ratio values with 95% confidence intervals (95% CI). This provides more accurate estimates compared with logit models when the binary outcome has a high prevalence [33,34].

To calculate SII and RII, individuals were ranked according to descending socioeconomic status (i.e. highest wealth or education level to lowest) to estimate their position in the cumulative distribution of socioeconomic status. The exposure variable can thus be interpreted as a continuous measure, with a value of zero equivalent to the top of the socioeconomic distribution and a value of one equivalent to the bottom. The SII and RII can be interpreted as the prevalence rate difference and the prevalence rate ratio, respectively, between those at top rank (representing the lowest level of education/wealth) and those at rank zero (representing the highest level of education/wealth), while taking into the effect across the entire distribution of education/wealth [35]. A SII value greater than zero and a RII value greater than one indicated higher risk factor prevalence among populations of lower socioeconomic status, or *regular* inequality (i.e. an inverse association between socioeconomic status and risk factor prevalence). Conversely, *reverse* inequality referred to higher risk factor prevalence among populations of higher socioeconomic position [36]. Data were adjusted for country of residence and age (Model 1), as well as other confounding factors: marital status, urban/rural area, and education or wealth (Model 2).

WHS had a stratified, multi-stage cluster design where each household had a known non-zero probability of selection. All analyses were weighted accounting for the individual survey sample designs. Specifically, each respondent in the country datasets was given a post-stratification sampling weight. This weight reflected each country’s population in such a way that if the sample size for two given countries are the same (but the population sizes of the countries are different), more weight is given to the country with higher population when calculating the pooled estimates. The non-independence of observations within the surveys clusters were also incorporated in the analysis using Taylor linearized variance estimation. Stata 11® was used for all analyses [37].

## Results

### Overall prevalence

Table 1 shows overall age-standardized prevalence of NCD risk factors in men and women living in study LMICs. Men reported higher prevalence than women for current daily smoking and heavy episodic alcohol drinking, and women had higher prevalence of physical inactivity. In both sexes, low fruit and vegetable consumption demonstrated the highest prevalence, with rates over 70%. With the exception of physical inactivity and heavy episodic alcohol drinking in women, the prevalence of each risk factor was significantly higher in MICs than LICs. Crude prevalence rates of NCD risk factors are shown in Additional file 4.

**Table 1:** Age-standardized prevalence of noncommunicable disease risk factors among adults aged 18 or higher of 48 low- and middle-income countries, World Health Survey 2002-04

		**Current daily smokers**			**Low-fruit/vegetable consumers**^a^			**Physically inactive people**^b^			**Heavy episodic alcohol drinkers**^c^		
		**Estimate**	** 95% CI**		**Estimate**	** 95% CI**		**Estimate**	** 95% CI**		**Estimate**	** 95% CI**	
**Men**	**Middle-income country group**	**34.1**	33.3	35.0	**79.1**	78.2	80.0	**12.3**	11.6	13.0	**12.6**	11.9	13.2
	**Low-income country group**	**25.2**	24.5	25.9	**72.8**	71.8	73.8	**8.4**	7.9	9.0	**6.9**	6.4	7.4
**Women**	**Middle-income country group**	**10.8**	10.2	11.3	**78.6**	77.7	79.5	**14.9**	14.2	15.5	**3.0**	2.8	3.3
	**Low-income country group**	**6.0**	5.6	6.4	**74.6**	73.7	75.5	**14.6**	14.0	15.3	**2.7**	2.3	3.0

Abbreviations: 95% CI, 95% Confidence Interval.

All numbers are in percentage.

^a^ No data were available for Mexico.

^b^ No data were available for Morocco and Latvia.

^c^ Mauritania; and Bosnia-Herzegovina, Comoros, Mauritania and Pakistan were excluded from males and females datasets, respectively.

### Wealth-related inequality

**Figure 1:**
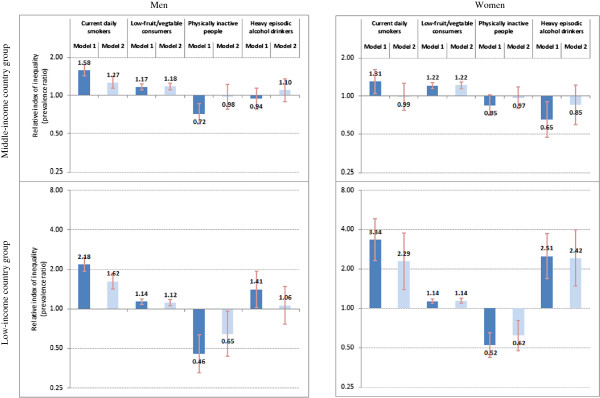
**Wealth-related relative inequality in noncommunicable disease risk factors among adults aged 18 or higher in 48 low- and middle-income countries, World Health Survey 2002-04.** The relative index of inequality shows wealth-related inequality in prevalence of current daily smoking, low fruit and vegetable consumption, physical inactivity, and heavy episodic alcohol drinking, among men and women aged 18 or higher, living in 48 low- and middle-income countries that participated in the 2002–04 World Health Survey. Individuals were cumulatively ranked by descending wealth quintiles, and prevalence ratios compared risk factor prevalence in the poorest to risk factor prevalence in the richest. Brackets indicate 95% confidence intervals. Model 1 data are adjusted for country of residence and age; Model 2 data are adjusted for country of residence, age, marital status, urban/rural area and education.

Table 2 summarizes age-standardized prevalence rates and absolute inequalities of NCD risk factors by household wealth quintile among men and women living in study LMICs. Relative inequalities are illustrated in Figure 1. (Additional file 5 summarizes crude prevalence of NCD risk factors by wealth among each sex-country income group.)

**Table 2:** Age-standardized prevalence of noncommunicable disease risk factors, by wealth quintile, and wealth-related absolute inequality among adults aged 18 or higher of 48 low- and middle-income countries, World Health Survey 2002-04

				**Current daily smokers**			**Low-fruit/ vegetable consumers**^a^			**Physically inactive people**^b^			**Heavy episodic alcohol drinkers**^c^		
				**Estimate**	** 95% CI**		**Estimate**	** 95% CI**		**Estimate**	** 95% CI**		**Estimate**	** 95% CI**	
**Men**	**Middle-income country group**	**Wealth quintile 1**		**43.6**	42.1	45.2	**83.8**	82.3	85.3	**11.6**	10.4	12.8	**14.3**	12.9	15.6
		**Wealth quintile 2**		**38.5**	37.0	40.0	**80.9**	79.5	82.4	**12.6**	11.5	13.7	**12.7**	11.5	13.8
		**Wealth quintile 3**		**34.7**	33.2	36.2	**79.7**	78.3	81.1	**11.7**	10.7	12.7	**12.6**	11.5	13.6
		**Wealth quintile 4**		**30.9**	29.5	32.3	**78.4**	77.0	79.8	**12.2**	11.1	13.2	**11.5**	10.5	12.5
		**Wealth quintile 5**		**27.9**	26.5	29.3	**75.2**	73.7	76.6	**13.5**	12.4	14.7	**12.5**	11.5	13.5
		**Slope index of inequality**	**Model 1**^d^	**13.3**	10.4	16.2	**11.6**	7.6	15.6	**−4.4**	−6.8	−1.9	**−0.8**	−3.4	1.8
			**Model 2**^e^	**6.9**	3.7	10.1	**12.0**	7.4	16.6	**−0.3**	−3.2	2.7	**1.3**	−1.5	4.1
	**Low-income country group**	**Wealth quintile 1**		**32.5**	31.1	33.9	**77.0**	75.5	78.6	**9.0**	8.0	10.0	**7.5**	6.6	8.3
		**Wealth quintile 2**		**28.6**	27.4	29.9	**74.1**	72.7	75.6	**7.8**	6.9	8.8	**6.7**	6.0	7.5
		**Wealth quintile 3**		**25.0**	23.8	26.2	**72.1**	70.6	73.5	**7.2**	6.3	8.1	**6.2**	5.5	7.0
		**Wealth quintile 4**		**22.1**	21.0	23.2	**71.8**	70.4	73.2	**8.3**	7.4	9.3	**6.9**	6.1	7.7
		**Wealth quintile 5**		**18.5**	17.4	19.6	**71.0**	69.6	72.5	**11.0**	9.9	12.1	**7.5**	6.7	8.3
		**Slope index of inequality**	**Model 1**^d^	**23.0**	19.6	26.4	**9.7**	6.2	13.2	**−5.0**	−7.1	−2.8	**1.2**	0.1	2.3
			**Model 2**^e^	**14.1**	10.2	18.0	**8.2**	4.3	12.1	**−2.7**	−5.2	−0.2	**0.2**	−0.9	1.4
**Women**	**Middle-income country group**	**Wealth quintile 1**		**13.2**	12.0	14.5	**82.9**	81.4	84.3	**14.4**	13.2	15.7	**2.6**	2.1	3.1
		**Wealth quintile 2**		**12.8**	11.7	13.9	**81.7**	80.4	83.0	**14.2**	13.2	15.2	**3.0**	2.5	3.5
		**Wealth quintile 3**		**10.8**	9.9	11.7	**79.7**	78.5	81.0	**14.5**	13.5	15.6	**2.8**	2.4	3.3
		**Wealth quintile 4**		**10.3**	9.3	11.2	**76.7**	75.4	78.1	**15.1**	14.1	16.2	**3.1**	2.6	3.5
		**Wealth quintile 5**		**8.8**	8.0	9.6	**73.2**	71.8	74.5	**15.7**	14.6	16.8	**3.3**	2.8	3.7
		**Slope index of inequality**	**Model 1**^d^	**2.3**	0.4	4.1	**14.4**	10.3	18.5	**−2.4**	−5.0	0.3	**−1.4**	−2.5	−0.3
			**Model 2**^e^	**−0.1**	−2.2	2.0	**14.7**	10.1	19.3	**−0.5**	−3.2	2.3	**−0.5**	−1.7	0.7
	**Low-income country group**	**Wealth quintile 1**		**8.8**	8.0	9.6	**77.8**	76.4	79.3	**13.3**	12.2	14.4	**2.5**	2.0	3.0
		**Wealth quintile 2**		**6.7**	6.0	7.3	**75.3**	73.9	76.7	**14.2**	13.1	15.3	**2.9**	2.3	3.5
		**Wealth quintile 3**		**5.9**	5.2	6.6	**73.2**	71.8	74.6	**13.7**	12.6	14.8	**2.8**	2.2	3.4
		**Wealth quintile 4**		**5.2**	4.6	5.7	**73.9**	72.5	75.2	**14.6**	13.5	15.7	**2.8**	2.2	3.3
		**Wealth quintile 5**		**3.0**	2.5	3.6	**72.4**	70.9	73.9	**18.2**	16.9	19.5	**2.6**	2.1	3.2
		**Slope index of inequality**	**Model 1**^d^	**5.7**	3.7	7.7	**9.5**	6.5	12.4	**−9.1**	−12.3	−5.9	**0.9**	0.5	1.4
			**Model 2**^e^	**3.8**	1.3	6.3	**9.9**	6.6	13.3	**−6.7**	−10.5	−2.9	**0.9**	0.3	1.4

Abbreviations: 95% CI, 95% Confidence Interval.

All numbers are in percentage. Wealth quintiles are ordered by increasing wealth.

^a^ No data were available for Mexico.

^b^ No data were available for Morocco and Latvia.

^c^ Mauritania; and Bosnia-Herzegovina, Comoros, Mauritania and Pakistan were excluded from male and female datasets, respectively.

^d^ Model 1 is adjusted for country of residence and age.

^e^ Model 2 is adjusted for country of residence, age, marital status, urban/rural area and education.

In both sexes, current daily smoking and low fruit and vegetable consumption were more prevalent in the poorest wealth quintile than in the richest, and regular inequality in both absolute and relative terms was found after controlling for respondents' age and country of residence (Model 1). The highest absolute inequality across the entire distribution of wealth was related to smoking among men living in LICs (prevalence difference: 23.0%, 95% CI: 19.6, 26.4). The absolute difference of low fruit and vegetable consumption prevalence between poorest and richest adults of the study LIC group was near 10% (Men: 9.7%, 95% CI: 6.2, 13.2; Women: 9.5%, 95% CI: 6.5, 12.4). SII values for smoking and low fruit and vegetable consumption were not significantly different in LIC and MIC study groups, except smoking in men, which demonstrated significantly higher absolute inequality in the LIC group.

On the contrary, physical inactivity illustrated reverse inequality, with elevated prevalence in populations of high socioeconomic status. Reverse inequality was pronounced in the LIC group. Inadequate physical activity among the poorest adults in LICs was about half as prevalent as among the richest (prevalence ratio: (Men) 0.46, 95% CI: 0.33, 0.64; (Women) 0.52, 95% CI: 0.42, 0.65).

Heavy episodic alcohol drinking demonstrated mixed patterns of inequality. For instance, women in LIC and MIC groups showed regular and reverse inequality, respectively (prevalence ratio: (LICs) 2.51, 95% CI: 1.68, 3.74; (MICs) 0.65, 95% CI: 0.47, 0.90), although the absolute inequality in both groups was very low (about 1 percentage point). No inequality was reported by men of the MIC group, and regular inequality was weakly demonstrated by men of the LIC group (prevalence ratio: 1.41, 95% CI: 1.03, 1.93).

Existing inequality was robust to further adjustments (Model 2) for most data.

### Education-related inequality

**Figure 2:**
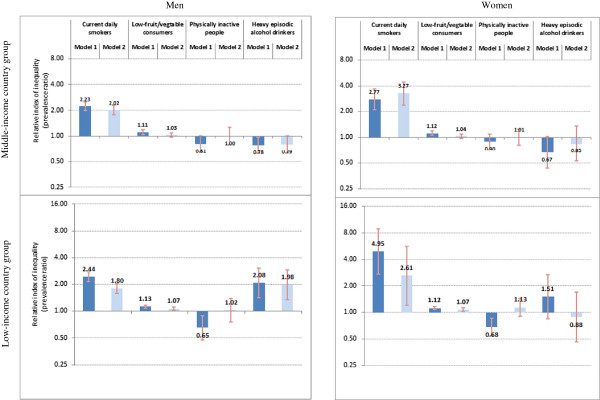
**Education-related relative inequality in non communicable disease risk factors among adults aged 18 or higher in 48 low- and middle-income countries, World Health Survey 2002-04.** The relative index of inequality shows education-related inequality in prevalence of current daily smoking, low fruit and vegetable consumption, physical inactivity, and heavy episodic alcohol drinking, among men and women aged 18 or higher, living in 48 low- and middle-income countries that participated in the 2002–04 World Health Survey. Individuals were cumulatively ranked by descending education level, and prevalence ratios compared risk factor prevalence in the least educated group to risk factor prevalence in the most educated group. Brackets indicate 95% confidence intervals. Model 1 data are adjusted for country of residence and age; Model 2 data are adjusted for country of residence, age, marital status, urban/rural area and wealth.

Table 3 summarizes age-standardized prevalence rates and absolute inequalities of NCD risk factors by educational level among men and women living in study LMICs. Relative inequalities are illustrated in Figure 2. (Additional file 6 summarizes crude prevalence of NCD risk factors by education among sex-country income groups.)

**Table 3:** Age-standardized prevalence of noncommunicable disease risk factors, by education level, and education-related absolute inequality among adults aged 18 or higher of 48 low- and middle-income countries, World Health Survey 2002-04

				**Current daily smokers**			**Low-fruit/vegetable consumers**^a^			**Physically inactive people**^b^			**Heavy episodic alcohol drinkers**^c^		
				**Estimate**	** 95% CI**		**Estimate**	** 95% CI**		**Estimate**	** 95% CI**		**Estimate**	** 95% CI**	
**Men**	**Middle-income country group**	**No formal schooling**		**40.0**	37.9	42.2	**82.0**	80.1	84.0	**18.9**	17.0	20.8	**9.9**	8.5	11.2
		**Less than primary school**		**36.7**	35.0	38.5	**82.3**	80.8	83.8	**16.2**	14.5	17.9	**10.2**	9.1	11.3
		**Primary school completed**		**37.8**	36.4	39.1	**80.3**	79.1	81.6	**12.3**	11.4	13.3	**13.7**	12.6	14.8
		**Secondary/high school completed**		**33.4**	32.3	34.5	**77.5**	76.3	78.6	**12.5**	11.6	13.4	**12.6**	11.8	13.5
		**College completed or above**		**21.8**	20.4	23.2	**75.6**	74.0	77.2	**14.7**	13.4	16.1	**11.6**	10.4	12.9
		**Slope index of inequality**	**Model 1**^d^	**24.0**	20.2	27.9	**8.0**	3.4	12.5	**−2.7**	−5.7	0.2	**−3.3**	−6.3	−0.3
			**Model 2**^e^	**20.8**	16.7	24.9	**2.3**	−2.5	7.1	**0.0**	−3.1	3.0	**−3.1**	−6.3	0.1
	**Low-income country group**	**No formal schooling**		**29.7**	28.3	31.1	**74.3**	72.8	75.8	**8.2**	7.4	9.0	**6.9**	6.0	7.9
		**Less than primary school**		**29.5**	28.1	31.0	**73.0**	71.6	74.5	**8.5**	7.4	9.7	**7.6**	6.8	8.4
		**Primary school completed**		**25.8**	24.5	27.2	**70.5**	69.0	72.1	**8.4**	7.3	9.4	**6.9**	6.1	7.7
		**Secondary/high school completed**		**19.8**	18.6	21.0	**72.9**	71.4	74.5	**10.0**	8.7	11.2	**7.5**	6.6	8.4
		**College completed or above**		**14.7**	13.2	16.3	**72.3**	70.3	74.4	**12.7**	11.2	14.1	**8.2**	6.9	9.6
		**Slope index of inequality**	**Model 1**^d^	**26.5**	22.8	30.3	**8.9**	5.7	12.0	**−2.7**	−4.7	−0.7	**2.7**	1.2	4.1
			**Model 2**^e^	**17.2**	13.3	21.1	**4.7**	1.6	7.8	**0.1**	−1.8	2.1	**2.5**	1.0	3.9
**Women**	**Middle-income country group**	**No formal schooling**		**10.8**	9.7	12.0	**85.8**	84.2	87.5	**22.8**	20.7	24.8	**2.3**	1.7	2.9
		**Less than primary school**		**10.6**	9.8	11.4	**82.6**	81.2	83.9	**21.2**	19.5	22.9	**2.0**	1.6	2.3
		**Primary school completed**		**13.6**	12.5	14.8	**81.6**	80.4	82.9	**15.1**	14.0	16.2	**3.7**	3.1	4.2
		**Secondary/high school completed**		**10.4**	9.8	11.1	**76.4**	75.2	77.6	**14.6**	13.8	15.5	**3.1**	2.7	3.5
		**College completed or above**		**8.4**	7.5	9.3	**74.1**	72.6	75.7	**14.9**	13.6	16.3	**3.1**	2.6	3.7
		**Slope index of inequality**	**Model 1**^d^	**8.9**	6.2	11.5	**8.5**	4.4	12.7	**−1.6**	−4.6	1.4	**−1.3**	−2.7	0.1
			**Model 2**^e^	**10.5**	7.4	13.6	**2.6**	−1.7	6.8	**0.2**	−2.9	3.2	**−0.5**	−2.1	1.0
	**Low-income country group**	**No formal schooling**		**7.3**	6.8	7.8	**76.0**	74.7	77.2	**14.0**	13.2	14.9	**2.4**	2.0	2.7
		**Less than primary school**		**5.6**	4.8	6.3	**73.6**	72.1	75.1	**13.9**	12.8	15.1	**1.9**	1.5	2.3
		**Primary school completed**		**2.3**	1.9	2.8	**72.0**	70.4	73.5	**15.1**	13.9	16.3	**3.1**	2.4	3.7
		**Secondary/high school completed**		**2.1**	1.6	2.7	**72.2**	70.4	73.9	**18.3**	16.9	19.8	**2.1**	1.4	2.7
		**College completed or above**		**1.4**	0.9	2.0	**69.5**	67.3	71.7	**16.0**	14.1	17.9	**2.8**	1.8	3.8
		**Slope index of inequality**	**Model 1**^d^	**7.1**	4.7	9.6	**8.5**	5.2	11.8	**−5.5**	−8.9	−2.1	**0.4**	−0.1	0.9
			**Model 2**^e^	**4.2**	1.1	7.3	**5.3**	1.8	8.9	**1.8**	−1.5	5.1	**−0.1**	−0.8	0.5

Abbreviations: 95% CI, 95% Confidence Interval.

All numbers are in percentage.

^a^ No data were available for Mexico.

^b^ No data were available for Morocco and Latvia.

^c^ Mauritania; and Bosnia-Herzegovina, Comoros, Mauritania and Pakistan were excluded from male and female datasets, respectively.

^d^ Model 1 is adjusted for country of residence and age.

^e^ Model 2 is adjusted for country of residence, age, marital status, urban/rural area and education.

Current daily smoking and low fruit and vegetable consumption showed regular inequality in both sexes and both country income groups. Smoking among adults with no education was at least twice as prevalent as among adults with college level or above, after controlling for age and country of residence. Absolute inequality in low fruit and vegetable consumption across the entire distribution of education was around 8% in both sexes and both country income groups.

After adjusting for age and country of residence, inequality in physical inactivity in the MIC group was not statistically significant, and reverse inequality was observed in the LIC group. In LICs, the prevalence of physical inactivity among people with no education was about two thirds of the rate of those with the highest education level.

Heavy episodic alcohol drinking data indicated mixed types of inequality, with reverse and regular inequalities among men of study MIC and LIC groups, respectively (prevalence difference: (MICs) -3.3%, 95% CI: -6.3, -0.3; (LICs) 2.7%, 95% CI: 1.2, 4.1). Similar trends for inequality patterns were observed for women of MIC and LIC groups, but values did not reach statistical significance (Model 1).

In most cases, observed inequalities remained significant after further adjustments. There was no inequality in physical inactivity in any group after data were adjusted for respondents' age and country of residence as well as marital status, area of residence, and wealth (Model 2).

## Discussion

The magnitude and direction of socioeconomic inequalities showed different patterns across risk factors, sex and country income group. Historically, the adoption of risky health behaviours tends to transition from higher to lower socioeconomic groups as countries grow richer: new behaviours are adopted earlier by higher socioeconomic groups who, over time, largely abandon these behaviours upon learning of the associated detrimental health effects (for example, due to health promotion efforts). Lower socioeconomic groups tend to engage in risky health behaviours later in the course of a country’s economic development [38]. Acknowledging that the timing and patterning of this transition are risk-factor dependent, these characteristic transition patterns may help to explain variance in our findings.

The strongest regular relative inequalities were reported for current daily smoking, particularly for education-related inequality. Other studies from developed, [36] and developing countries [39,40] have also reported significant education-based disparities in smoking rates, and the importance of tobacco control in LMICs has been widely recognized as an immediate public health priority [5,41,42]. These findings underscore the importance of integrating equity considerations into widespread tobacco control efforts, including planning, implementation, monitoring and surveillance activities. The *WHO Framework Convention on Tobacco Control*, [43] the primary tool developed by and available to countries to control tobacco use and exposure, noted the serious burden that the production and consumption of tobacco products place on the poor. While tobacco reduction efforts have achieved considerable success in high-income countries, [44,45] tobacco companies have intensified marketing strategies to target vulnerable populations of LMICs, such as women and adolescents [46-48]. Interventions at national and international levels stand to benefit by adopting equity focused approaches to reduce smoking prevalence, bearing in mind that population groups may differ in their ability to participate in such initiatives and/or experience intended health benefits [49-51].

Low fruit and vegetable consumption demonstrated regular inequality, with consistently higher prevalence among the disadvantaged across all study groups. Hall et al. [23] were the first to quantify socio-demographic prevalence data for fruit and vegetable consumption in 52 WHS countries, also noting a trend for decreased risk factor prevalence with increased household wealth status, and prevalence rates of low fruit and vegetable consumption in excess of 70%. The widespread nature of low fruit and vegetable consumption indicates an opportunity for population-wide interventions. At the same time, observed regular inequality calls attention to the need for equity-based approaches targeting populations of lower socioeconomic standing. Given the shortage of data about fruit and vegetable consumption in many LMICs, [52] additional research is warranted at national and international levels to further characterize these trends, with intensified efforts to collect these data with the necessary precision across all socioeconomic strata.

Our measure of physical inactivity did not differentiate between physical activity at work, commuting or during leisure time, distinctions which may demonstrate diverging patterns according to socioeconomic position. Leisure-time physical activity has been reported to increase with socioeconomic position, while work-time physical activity tended to decrease [53,54]. Because leisure-time activity has been found to constitute a relatively small proportion of overall physical activity in LMICs, [55,56] the socioeconomic gradients observed in the present study may reflect high levels of physical activity associated with work or commuting among those of lower education or household income levels. The promotion of physical activity as an aspect of NCD prevention in LMICs has been largely neglected; increased advocacy and partnerships across health and non-health sectors, however, can help to create and maintain physical, social and cultural environments that encourage active lifestyles [57].

For heavy episodic alcohol drinking, data indicated a mixed pattern of inequality, as has been reported by other cross-national studies in countries of all income levels [58]. Harmful use of alcohol is the third leading risk factor for global disease burden, [59] with complex determinants including cultural practices, lifestyle and social status influences on one hand, and health risk considerations on the other [60]. NCD prevention efforts should work both nationally and globally to target the general population as well as subpopulations with a greater chance of engaging in heavy episodic drinking. The WHO global strategy to reduce the harmful use of alcohol outlines action-oriented strategies for implementation at global, regional, sub-regional and national levels [61].

Overall, wealth- and education-related inequalities showed similar directional trends for each risk factor, with varying magnitude across sex-country income groups, indicating a concerted need for educational and poverty-reduction oriented policies. Poverty-reduction strategies may contribute to the success of policies addressing risk factors that displayed prominent within-country regular, wealth-based inequality, such as current daily smoking, low fruit and vegetable consumption and, within LICs, heavy episodic alcohol drinking. Kim et al. [62] highlighted the importance of using education to establish cultural norms that reinforce healthier lifestyles. This approach may be particularly effective in situations demonstrating strong regular education-based inequality. While education grouping was on an absolute basis-- i.e. the highest level of education attainment, irrespective of country of residence-- wealth quintiles were derived from the asset index, which scored the relative position of each household comparing to others within each country. We showed fairly similar patterning of risk factors across education and wealth levels, despite that these stratifiers were defined based on different terms (absolute or relative). In other words, wealth- and education-related inequalities in health exist in countries at all levels of development. The present study did not explore possible interactions between wealth- and education-related health inequalities. Thus, it remains unknown whether a reduction in education-based inequality, for example, would impact wealth-based health inequalities.

### Strengths and limitations

This study quantified and compared wealth-and education-based inequality in four health risk factors, stratified by sex and country-income group. The resolution of the *United Nations High-level Meeting on NCDs*[63] recognized the disaggregation and analysis of data as an important component of strengthening national policies and health systems. Combining national level datasets facilitated broad-scale analyses of socioeconomic distribution of leading NCD risk factors, contributing international evidence from understudied LMIC regions. Wealth classification by quintiles is an accepted method of making relative comparisons among respondents, although patterns of wealth distribution may vary between countries. Likewise, education levels were standardized to be comparable across countries.

Pooling datasets inherently masks individual country differences; however, a country variable was included in the multivariate model in order to control for any potential confounding effect related to the individual countries.

It is possible that a selection bias may have occurred in the sampling process especially in countries with lower response rates, although we are not aware of evidence to suggest that this had occurred. The main reason for household non-response was inability to locate the selected households, or the households refusing to participate even before a roster could be obtained.

The study countries were not probabilistically selected and therefore are not necessarily representative of all LMICs.

During analysis and discussion the health impact of the variable level of risk by each risk factor was not explored. A comparable level of alcohol consumption has been shown to result in higher levels of alcohol-attributed harm among poorer populations, [60] and within certain geographical regions [64]. Measuring prevalence alone may not fully represent inequalities in the impact of the studied health risks.

Finally, this is a cross-sectional study that examined inequalities in the distribution of risk factors across a range of countries at a point in time. Patterns of distribution of risk factors, however, may fluctuate over time, with variable increases, decreases, or periods of stagnation across sections of a population (as was evident for example, with smoking rates in high income countries [65]). Thus, longitudinal studies are needed to track these changes over time and to understand the drivers of these trends. Other NCD risk factors, such as blood glucose or lipid levels, were not included in the present study, but may show inequalities in their distribution. Future studies may include a broader range of risk factors to provide a more comprehensive distribution of risk profiles across socioeconomic groups.

## Conclusions

The recent political declaration from the *United Nations High-level Meeting on NCDs* recognized that poverty, uneven distribution of wealth and lack of education, as well as economic, social, gender, political, behavioral and environmental determinants of health, contribute to the rising incidence and prevalence of NCDs. The declaration called for monitoring of exposure to risk factors and their social and economic determinants in order to appropriately address NCDs [63]. Prevalence data from four NCD risk factors revealed varying degrees of socioeconomic inequality in LIC and MIC settings. The *WHO 2008–2013 Action Plan for the Global Strategy for the Prevention and Control of Noncommunicable Diseases*[4] recommended a comprehensive approach to risk factor reduction, combining population-wide health promotion with efforts targeted to those at higher risk.

Rose (1985) suggested complementary approaches to preventive health actions, centered around the high-risk strategy and the population strategy [66]. A high-risk strategy impedes risk distribution by targeting resources to high-risk individuals who are most likely to partake in a risky health behavior, thus enhancing the cost effectiveness of preventive health programs, improving the benefit to risk ratio, and increasing the likelihood for appropriate interventions. For example, current daily smoking and low fruit and vegetable consumption consistently demonstrated regular inequality, emphasizing the importance of equity-focused policy and program approaches. Integrating equity components into monitoring and surveillance is a step towards ensuring that interventions reach and benefit high-risk populations [9].

Rose’s population strategy prescribes radical changes at a population level, attempting sweeping environmental changes and shifting behavioral norms [66]. Effective poverty-reduction and education-based campaigns will help to improve conditions that enable better health outcomes at a population level [67].

## Abbreviations

95% CI: 95% confidence interval; LICs: Low-income countries; LMICs: Low- and middle-income countries; MICs: Middle-income countries; NCDs: Non communicable diseases; RII: Relative index of inequality; SII: Slope index of inequality; WHO: World Health Organization; WHS: World Health Survey.

## Competing interests

The authors declare that they have no competing interests.

## Authors' contributions

AH designed the study with inputs from AK. AH did the statistical analysis with inputs from RG, SH and NN. NB wrote the first draft with inputs from AH. SC, SH, RG, AK, NN, DR and ETE read the draft and provided critical comments. All authors read and approved the final draft.

## Pre-publication history

The pre-publication history for this paper can be accessed here:

http://www.biomedcentral.com/1471-2458/12/912/prepub

## Supplementary Material

Additional file 1 **Title.** Study sample size, by country and sex, World Health Survey 2002–04. Description: Displays the study sample size of men and women (aged 18 or higher) from 48 low- and middle-income countries that participated in the 2002–04 World Health Survey. Click here for file

Additional file 2 **Title.** Non-response rates of risk factors for noncommunicable diseases, by country income group and sex, World Health Survey 2002–04. Description: Displays the non-response rates to World Health Survey individual questionnaires for each studied noncommunicable disease risk factor, grouped by sex and low- or middle-income country status. Data represent 48 low- and middle-income countries that participated in the 2002–04 World Health Survey. Click here for file

Additional file 3 **Title.** List of in-country collaborating partners (local review boards). Click here for file

Additional file 4 **Title.** Crude prevalence of risk factors for noncommunicable diseases among adults aged 18 or higher living in 48 low- and middle-income countries, World Health Survey 2002–04. Description: Displays the crude prevalence rates (percentage) and 95% confidence intervals for each studied noncommunicable disease risk factor among adults (aged 18 or higher), grouped by sex and low- or middle-income country status. Data represent 48 low- and middle-income countries that participated in the 2002–04 World Health Survey. Click here for file

Additional file 5 **Title.** Crude prevalence of risk factors for noncommunicable diseases among adults aged 18 or higher living in 48 low- and middle-income countries, by wealth, World Health Survey 2002–04. Description: Displays the crude prevalence rates (percentage) and 95% confidence interval for each studied noncommunicable disease risk factor among adults (aged 18 or higher), according to wealth quintile. Data are grouped by sex and low- or middle-income country status, and represent 48 low- and middle-income countries that participated in the 2002–04 World Health Survey. Click here for file

Additional file 6 **Title.** Crude prevalence of risk factors for noncommunicable diseases among adults aged 18 or higher living in 48 low- and middle-income countries, by education level, World Health Survey 2002–04. Description: Displays the crude prevalence rates (percentage) and 95% confidence interval for each studied noncommunicable disease risk factor among adults (aged 18 or higher), according to education level. Data are grouped by sex and low- or middle-income country status, and represent 48 low- and middle-income countries that participated in the 2002–04 World Health Survey. Click here for file
