# Supplementary material for: Socioeconomic inequalities in risk factors for non communicable diseases in low-income and middle-income countries: results from the World Health Survey
Source: BMC Public Health. 2012 Oct 28;12:912. doi: 10.1186/1471-2458-12-912 (PMC3507902; doi:10.1186/1471-2458-12-912)
Supplement: Additional file 1 — Title. Study sample size, by country and sex, World Health Survey 2002–04. Description: Displays the study sample size of men and women (aged 18 or higher) from 48 low- and middle-income countries that participated in the 2002–04 World Health Survey. [file 1471-2458-12-912-S1.pdf]

Additional file 1. Study sample size, by country and sex, World Health Survey 2002-04

**Middle-income countries**	**Total**	**Men**	**Women**	**Low-income countries**	**Total**	**Men**	**Women**
Bosnia and Herzegovina	1,028	434	594	Bangladesh	5,550	2,584	2,966
Brazil	5,000	2,188	2,812	Burkina Faso	4,821	2,271	2,550
China	3,993	1,954	2,039	Chad	4,635	2,191	2,444
Croatia	990	401	589	Comoros	1,758	786	972
Czech Republic	935	419	516	Congo	2,487	1,164	1,323
Dominican Republic	4,534	2,104	2,430	Cote d'Ivoire	3,178	1,815	1,363
Ecuador	4,608	2,043	2,565	Ethiopia	4,936	2,390	2,546
Estonia	1,011	367	644	Ghana	3,931	1,772	2,159
Georgia	2,749	1,162	1,587	India	9,723	4,705	5,018
Hungary	1,419	591	828	Kenya	4,346	1,847	2,499
Kazakhstan	4,496	1,544	2,952	Lao People's Democratic Republic	4,888	2,295	2,593
Latvia	855	286	569	Malawi	5,297	2,210	3,087
Malaysia	6,038	2,671	3,367	Mali	4,145	2,382	1,763
Mauritius	3,888	1,872	2,016	Mauritania	3,705	1,440	2,265
Mexico	38,617	16,327	22,290	Myanmar	5,886	2,551	3,335
Morocco	4,472	2,074	2,398	Nepal	8,686	3,698	4,988
Namibia	4,246	1,722	2,524	Pakistan	6,104	3,402	2,702
Paraguay	5,131	2,348	2,783	Senegal	2,970	1,548	1,422
Philippines	10,075	4,659	5,416	Viet Nam	3,491	1,571	1,920
Russian Federation	4,422	1,593	2,829	Zambia	3,810	1,722	2,088
Slovakia	2,488	953	1,535	Zimbabwe	4,061	1,477	2,584
South Africa	2,351	1,115	1,236				
Sri Lanka	6,698	3,117	3,581				
Swaziland	3,061	1,397	1,664				
Tunisia	5,068	2,343	2,725				
Ukraine	2,498	883	1,615				
Uruguay	2,977	1,447	1,530				
**Total**	**133,648**	**58,014**	**75,634**	**Total**	**98,408**	**45,821**	**52,587**
