# Supplementary material for: Socioeconomic inequalities in risk factors for non communicable diseases in low-income and middle-income countries: results from the World Health Survey
Source: BMC Public Health. 2012 Oct 28;12:912. doi: 10.1186/1471-2458-12-912 (PMC3507902; doi:10.1186/1471-2458-12-912)
Supplement: Additional file 2 — Title. Non-response rates of risk factors for noncommunicable diseases, by country income group and sex, World Health Survey 2002–04. Description: Displays the non-response rates to World Health Survey individual questionnaires for each studied noncommunicable disease risk factor, grouped by sex and low- or middle-income country status. Data represent 48 low- and middle-income countries that participated in the 2002–04 World Health Survey. [file 1471-2458-12-912-S2.pdf]

Additional file 2. Non-response rates of risk factors for noncommunicable diseases, by country income group and sex, World Health Survey 2002-04

		Current daily smokers	Low-fruit/vegetable consumers [a]	Physically inactive people [b]	Heavy episodic alcohol drinkers [c]
**Men**	**Middle-income country group**	2.1	8.7	4.6	2.8
	**Low-income country group**	1.5	11.9	5.3	3.1
**Women**	**Middle-income country group**	2.1	8.8	4.1	2.4
	**Low-income country group**	1.3	10.6	4.8	1.9

All numbers are in percentage
[a] No data were available for Mexico
[b] No data were available for Morocco and Latvia
[c] Mauritania; and Bosnia-Herzegovina, Comoros, Mauritania and Pakistan were excluded from males and females datasets, respectively
