# Supplementary material for: Socioeconomic inequalities in risk factors for non communicable diseases in low-income and middle-income countries: results from the World Health Survey
Source: BMC Public Health. 2012 Oct 28;12:912. doi: 10.1186/1471-2458-12-912 (PMC3507902; doi:10.1186/1471-2458-12-912)
Supplement: Additional file 3 — Title. List of in-country collaborating partners (local review boards). [file 1471-2458-12-912-S3.pdf]

Additional file 3. List of in-country collaborating partners (local review boards)

Country	Country focal point
Bangladesh	Mitra and Associates
Bosnia and Herzegovina	The Federal Public Health Institute
Brazil	Fundacao Oswaldo Cruz
Burkina Faso	Institut de Recherche en Sciences de la Santé
Chad	Faculté des Sciences de la Santé, Univ N'Djamena
China	Centre for Health Statistics Information
Comoros	Bureau Comorien de Conseil
Congo	Unité de recherche sur les systèmes de santé
Cote d'Ivoire	Ministère de la Santé
Croatia	The Croatian National Institute of Public Health
Czech Republic	Institute of Health Information and Statistics
Dominican Republic	Centro de Estudios Sociales y Demográficos (CESDEM)
Ecuador	Fundación Ecuatoriana para la Salud y el Desarroll (FESALUD)
Estonia	Saar Poll Ltd.
Ethiopia	Department of Community Health, Jimma University
Georgia	Georgian State Medical Academy (GSMA)
Ghana	Department of Community Health, Ghana Medical School
Hungary	Johan Bela National Centre for Epidemiology
India	International Institute of Population Sciences
Kazakhstan	Kazakhstan School of Public Health (KSPH)
Kenya	Central Bureau of Statistics
Lao People's Democratic Republic	National Institute of Public Health, Ministry of Health
Latvia	The Health Promotion Center
Malawi	Centre for Social Research (CSR)
Malaysia	Public Health Institute, Ministry of Health
Mali	Cellule de Planification et de Statistique, (CPS)
Mauritania	Office Nationale de la Statistique (ONS)
Mauritius	Mauritius Institute of Health
Mexico	Instituto Nacional de Salud Pública
Morocco	Ministère de la Santé
Myanmar	Department of Medical Research, Ministry of Health
Namibia	Ministry of Health
Nepal	ORG-MARG Nepal PVT.Ltd
Pakistan	Ministry of Health
Paraguay	Fac.de Ciencias Veterinarias, Univ. Nacional/DGEEC
Philippines	College of Medicine, University of the Philippines
Russian Federation	Semashko Institute for Research on Social Hygiene
Senegal	Direction Etudes, Recherche et Formation (DERF)
Slovakia	Environment,s.c., Centre of Biostatistics and Envi
South Africa	Community Agency for Social Enquiry (CASE)
Sri Lanka	Ministry of Health
Swaziland	Faculty of Health Sciences, Univ Swaziland
Tunisia	Institut National de la Santé Publique
Ukraine	Odessa State Medical University
Uruguay	Centro de Estudios de Economia y Salud (CEES)
Viet Nam	Ministry of Health
Zambia	School of Humanities & Social Sciences, University of Zambia
Zimbabwe	Community Health, University of Zimbabwe
