# Supplementary material for: Socioeconomic inequalities in risk factors for non communicable diseases in low-income and middle-income countries: results from the World Health Survey
Source: BMC Public Health. 2012 Oct 28;12:912. doi: 10.1186/1471-2458-12-912 (PMC3507902; doi:10.1186/1471-2458-12-912)
Supplement: Additional file 4 — Title. Crude prevalence of risk factors for noncommunicable diseases among adults aged 18 or higher living in 48 low- and middle-income countries, World Health Survey 2002–04. Description: Displays the crude prevalence rates (percentage) and 95% confidence intervals for each studied noncommunicable disease risk factor among adults (aged 18 or higher), grouped by sex and low- or middle-income country status. Data represent 48 low- and middle-income countries that participated in the 2002–04 World Health Survey. [file 1471-2458-12-912-S4.pdf]

Additional file 4. Crude prevalence of risk factors for noncommunicable diseases among adults aged 18 or higher living in 48 low- and middle-income countries, World Health Survey 2002-04

		**Current daily smokers**			**Low-fruit/vegetable consumers [a]**			**Physically inactive people [b]**			**Heavy episodic alcohol drinkers [c]**		
		**Estimate**	**95%CI**		**Estimate**	**95%CI**		**Estimate**	**95%CI**		**Estimate**	**95%CI**	
**Men**	**Middle-income country group**	**29.0**	28.0	29.9	**73.7**	72.1	75.2	**13.0**	12.1	14.0	**13.6**	12.7	14.4
	**Low-income country group**	**29.7**	28.3	31.0	**74.8**	73.0	76.7	**6.3**	5.5	7.1	**3.5**	3.1	4.0
**Women**	**Middle-income country group**	**8.5**	7.9	9.0	**74.0**	72.6	75.5	**14.1**	13.2	15.0	**3.3**	2.9	3.6
	**Low-income country group**	**4.7**	4.2	5.3	**74.7**	72.9	76.5	**14.1**	13.0	15.1	**1.0**	0.8	1.2

Abbreviations: 95% CI, 95% Confidence Interval

All numbers are in percentage

[a] No data were available for Mexico

[b] No data were available for Morocco and Latvia

[c] Mauritania; and Bosnia-Herzegovina, Comoros, Mauritania and Pakistan were excluded from males and females datasets, respectively
