# Supplementary material for: Socioeconomic inequalities in risk factors for non communicable diseases in low-income and middle-income countries: results from the World Health Survey
Source: BMC Public Health. 2012 Oct 28;12:912. doi: 10.1186/1471-2458-12-912 (PMC3507902; doi:10.1186/1471-2458-12-912)
Supplement: Additional file 5 — Title. Crude prevalence of risk factors for noncommunicable diseases among adults aged 18 or higher living in 48 low- and middle-income countries, by wealth, World Health Survey 2002–04. Description: Displays the crude prevalence rates (percentage) and 95% confidence interval for each studied noncommunicable disease risk factor among adults (aged 18 or higher), according to wealth quintile. Data are grouped by sex and low- or middle-income country status, and represent 48 low- and middle-income countries that participated in the 2002–04 World Health Survey. [file 1471-2458-12-912-S5.pdf]

Additional file 5. Crude prevalence of risk factors for noncommunicable diseases among adults aged 18 or higher living in 48 low- and middle-income countries, by wealth, World Health Survey 2002-04

			Current daily smokers			Low-fruit/vegetable consumers [a]			Physically inactive people [b]			Heavy episodic alcohol drinkers [c]		
			**Estimate**	**95% CI**		**Estimate**	**95% CI**		**Estimate**	**95% CI**		**Estimate**	**95% CI**	
**Men**	**Middle-income country group**	**Wealth quintile 1**	**33.6**	31.3	35.8	**79.9**	77.3	82.4	**9.9**	8.2	11.5	**13.0**	11.3	14.6
		**Wealth quintile 2**	**31.7**	29.7	33.7	**76.5**	73.6	79.4	**13.8**	11.7	16.0	**13.4**	11.8	15.0
		**Wealth quintile 3**	**29.4**	27.5	31.2	**74.5**	71.9	77.1	**13.9**	11.9	15.9	**13.4**	11.9	15.0
		**Wealth quintile 4**	**27.1**	25.3	29.0	**72.4**	69.7	75.1	**13.7**	11.9	15.4	**13.6**	12.0	15.3
		**Wealth quintile 5**	**24.5**	22.8	26.1	**67.6**	65.1	70.1	**13.4**	11.8	15.0	**14.3**	12.6	15.9
	**Low-income country group**	**Wealth quintile 1**	**40.2**	37.6	42.8	**80.0**	77.3	82.7	**5.5**	4.2	6.7	**4.5**	3.6	5.5
		**Wealth quintile 2**	**35.7**	33.3	38.2	**77.9**	75.2	80.6	**4.5**	3.4	5.5	**3.6**	2.9	4.2
		**Wealth quintile 3**	**29.6**	27.0	32.3	**76.2**	73.5	78.9	**6.2**	4.5	8.0	**3.0**	2.4	3.5
		**Wealth quintile 4**	**24.9**	22.8	27.0	**71.9**	69.4	74.4	**6.2**	4.8	7.5	**3.3**	2.7	3.8
		**Wealth quintile 5**	**20.3**	18.3	22.3	**69.7**	66.8	72.6	**8.8**	7.4	10.3	**3.6**	2.7	4.4
**Women**	**Middle-income country group**	**Wealth quintile 1**	**8.9**	7.6	10.2	**80.1**	77.5	82.7	**13.9**	11.9	15.9	**2.3**	1.7	2.9
		**Wealth quintile 2**	**8.5**	7.4	9.5	**79.5**	77.5	81.5	**13.8**	12.3	15.3	**2.9**	2.3	3.5
		**Wealth quintile 3**	**8.8**	7.7	9.9	**75.9**	73.7	78.1	**14.5**	12.9	16.1	**3.6**	2.8	4.4
		**Wealth quintile 4**	**8.4**	7.2	9.5	**70.6**	68.0	73.2	**12.9**	11.5	14.3	**3.5**	2.8	4.3
		**Wealth quintile 5**	**7.8**	6.7	8.9	**66.8**	64.1	69.6	**15.0**	13.3	16.8	**3.8**	3.0	4.5
	**Low-income country group**	**Wealth quintile 1**	**7.2**	5.4	9.0	**78.9**	76.2	81.6	**11.5**	9.8	13.2	**1.3**	0.9	1.6
		**Wealth quintile 2**	**5.5**	4.6	6.5	**78.2**	75.8	80.6	**13.1**	11.3	14.9	**1.3**	0.9	1.7
		**Wealth quintile 3**	**5.4**	4.2	6.5	**73.0**	70.4	75.6	**12.6**	10.9	14.3	**0.9**	0.6	1.2
		**Wealth quintile 4**	**3.7**	2.9	4.5	**72.7**	70.1	75.4	**14.4**	12.4	16.4	**0.8**	0.5	1.0
		**Wealth quintile 5**	**2.0**	1.4	2.6	**71.3**	68.8	73.7	**18.9**	16.7	21.2	**0.7**	0.5	0.8

Abbreviations: 95% CI, 95% Confidence Interval
All numbers are in percentage
[a] No data were available for Mexico
[b] No data were available for Morocco and Latvia
[c] Mauritania; and Bosnia-Herzegovina, Comoros, Mauritania and Pakistan were excluded from males and females datasets, respectively
