# Supplementary material for: Socioeconomic inequalities in risk factors for non communicable diseases in low-income and middle-income countries: results from the World Health Survey
Source: BMC Public Health. 2012 Oct 28;12:912. doi: 10.1186/1471-2458-12-912 (PMC3507902; doi:10.1186/1471-2458-12-912)
Supplement: Additional file 6 — Title. Crude prevalence of risk factors for noncommunicable diseases among adults aged 18 or higher living in 48 low- and middle-income countries, by education level, World Health Survey 2002–04. Description: Displays the crude prevalence rates (percentage) and 95% confidence interval for each studied noncommunicable disease risk factor among adults (aged 18 or higher), according to education level. Data are grouped by sex and low- or middle-income country status, and represent 48 low- and middle-income countries that participated in the 2002–04 World Health Survey. [file 1471-2458-12-912-S6.pdf]

Additional file 6. Crude prevalence of risk factors for noncommunicable diseases among adults aged 18 or higher living in 48 low- and middle-income countries, by education, World Health Survey 2002-04

			Current daily smokers			Low-fruit/vegetable consumers [a]			Physically inactive people [b]			Heavy episodic alcohol drinkers [c]		
			Estimate	95% CI		Estimate	95% CI		Estimate	95% CI		Estimate	95% CI	
**Men**	**Middle-income country group**	**No formal schooling**	**36.2**	32.9	39.5	**84.6**	81.7	87.5	**19.2**	15.2	23.1	**6.1**	4.4	7.9
		**Less than primary school**	**32.7**	29.9	35.6	**74.1**	71.2	77.1	**17.1**	14.4	19.9	**12.6**	10.7	14.5
		**Primary school completed**	**31.1**	29.1	33.1	**75.7**	73.7	77.7	**14.5**	12.6	16.4	**14.2**	12.5	15.9
		**Secondary/high school completed**	**26.5**	25.3	27.8	**72.0**	69.9	74.2	**12.0**	11.0	13.0	**13.8**	12.8	14.9
		**College completed or above**	**29.6**	27.0	32.2	**72.1**	68.7	75.5	**10.7**	8.5	13.0	**15.7**	13.4	18.0
	**Low-income country group**	**No formal schooling**	**38.5**	36.2	40.9	**79.3**	77.0	81.7	**6.9**	5.5	8.3	**3.7**	3.0	4.4
		**Less than primary school**	**35.2**	32.6	37.9	**70.2**	67.2	73.1	**4.2**	3.3	5.1	**4.7**	3.9	5.5
		**Primary school completed**	**28.1**	26.2	30.0	**71.2**	68.6	73.7	**5.8**	4.6	7.0	**3.1**	2.6	3.6
		**Secondary/high school completed**	**22.2**	20.5	24.0	**76.0**	73.6	78.5	**6.5**	5.2	7.7	**3.3**	2.6	4.1
		**College completed or above**	**15.8**	12.8	18.9	**73.5**	69.9	77.2	**8.2**	6.0	10.5	**2.7**	1.1	4.4
**Women**	**Middle-income country group**	**No formal schooling**	**9.3**	7.4	11.2	**88.3**	86.3	90.2	**24.4**	20.6	28.2	**1.4**	0.7	2.1
		**Less than primary school**	**13.3**	11.2	15.5	**76.8**	74.0	79.6	**20.9**	17.7	24.1	**3.2**	2.2	4.1
		**Primary school completed**	**10.4**	9.1	11.7	**74.1**	71.9	76.3	**14.9**	13.2	16.6	**3.2**	2.4	3.9
		**Secondary/high school completed**	**7.0**	6.4	7.6	**72.1**	70.2	74.1	**13.2**	12.2	14.2	**3.3**	2.9	3.8
		**College completed or above**	**7.4**	6.1	8.8	**71.1**	68.1	74.2	**9.3**	7.6	10.9	**4.3**	3.4	5.2
	**Low-income country group**	**No formal schooling**	**7.1**	6.2	8.0	**79.7**	77.6	81.8	**15.4**	14.0	16.7	**1.5**	1.2	1.9
		**Less than primary school**	**4.4**	3.4	5.3	**67.7**	65.0	70.4	**11.8**	10.1	13.5	**0.6**	0.4	0.8
		**Primary school completed**	**1.9**	1.2	2.5	**65.4**	62.7	68.0	**12.3**	10.5	14.0	**0.5**	0.3	0.7
		**Secondary/high school completed**	**1.4**	0.5	2.4	**75.5**	72.6	78.4	**13.3**	11.2	15.5	**0.5**	0.2	0.9
		**College completed or above**	**0.4**	0.1	0.7	**77.2**	73.0	81.4	**15.9**	12.0	19.9	**0.5**	0.1	0.9

Abbreviations: 95% CI, 95% Confidence Interval

All numbers are in percentage

[a] No data were available for Mexico

[b] No data were available for Morocco and Latvia

[c] Mauritania; and Bosnia-Herzegovina, Comoros, Mauritania and Pakistan were excluded from males and females datasets, respectively
